# Supplementary material for: Are carbon emissions trading and green financial instruments synergistic? -Comprehensive quantitative research based on content analysis
Source: PLoS One. 2024 Mar 7;19(3):e0298601. doi: 10.1371/journal.pone.0298601 (PMC10919601; doi:10.1371/journal.pone.0298601)
Supplement: S1 Data — (ZIP) [file pone.0298601.s001.zip › Data/地级市Z_F工作报告.zip!/║┌┴·╜¡/╣■╢√▒⌡╩╨/└┤╫╘╣1⁄2╓┌║┼í╛┬φ┐╦╩2╛▌═°í┐_2011.docx]

各位代表：

　　现在，我代表市人民政府向大会报告工作，请予审议，并请市政协各位委员提出意见。

　　一、“十一五”时期简要回顾及2010年工作情况

　　“十一五”时期是我市发展史上极不平凡的5年。5年来，在省委、省政府和市委的正确领导下，市政府紧紧依靠全市人民，深入贯彻落实科学发展观，积极应对国际金融危机等不利因素影响，全力推动经济社会又好又快发展，圆满完成“十一五”规划确定的各项任务，其中地区生产总值、地方财政收入、全社会固定资产投资和污染减排指标提前1年实现目标。

　　———结构调整优化升级，综合实力显著增强。“十一五”时期，全市地区生产总值从1796.4亿元增加到3665.9亿元，年均增长13.5%；人均地区生产总值从2790美元增加到5583美元，年均增长13%；地方财政一般预算收入从98亿元增加到238.1亿元，年均增长19.4%；固定资产投资5年累计达7725.8亿元，是“十五”时期的3.4倍，年均增长32.9%；社会消费品零售总额累计达6488.2亿元，是“十五”时期的2倍，年均增长17.6%。万元GDP综合能耗累计下降21%。三次产业比重由2005年的15.5:34.3:50.2调整为11.3:37.8:50.9。农业基础地位更加巩固，综合生产能力大幅提升，5年累计生产粮食1081亿斤，是“十五”时期的1.4倍；工业主导作用进一步增强，装备制造、食品、医药、石化4大优势产业增加值占规模以上工业比重达83.5%；服务业层次和水平有新提高，成为国家服务业综合改革试点区域。所有制结构不断优化，非公有制经济增加值从861亿元增加到1904.3亿元，年均增长15%，占地区生产总值比重达51.9%。高新技术产业产值从718亿元增加到2042亿元，年均增长23.1%，科技进步对经济增长贡献率由41.7%提高到53%，连续获得全国科技进步先进城市称号，成为首批国家创新型试点城市。

　　———城乡建设步伐加快，载体功能日臻完善。累计完成城市基础设施投资2760.2亿元，是“十五”时期的3.3倍；地铁一号线、哈西客站等重大基础设施项目顺利推进，松北、群力、哈西等新区建设取得积极进展，磨盘山供水、天然气置换等一批重大工程全面完成。新增集中供热面积1164万平方米，集中供热普及率达67%。市区环境空气质量2级及2级以上天数达到国家环保模范城标准。松花江出境水质主要污染指标累计下降12.8%。绕城高速公路全线贯通。新改建农村公路1.4万公里，是“十五”时期的11倍，高等级路面农村公路通乡、通村率实现100%。

　　———改革开放不断深化，发展动力和活力持续增强。国有企业改制基本完成，厂办大集体改制全面启动，政府机构改革、财税体制改革、农村综合改革以及医药卫生、文化教育、公共服务等领域改革取得积极成果。累计实际利用外资27.1亿美元，年均增长16.2%；累计国内经济技术合作资金到位额1546亿元，年均增长38%；累计实现进出口贸易额163.7亿美元，年均增长15.6%。对外交往取得新进展，累计与25个国家的30个城市缔结友好城市关系。

　　———社会事业全面进步，人民群众得到更多实惠。坚持实施惠民行动，促进重点民生问题有效解决。城市居民人均可支配收入由10065元增加到17557元，比2005年增长74.4%；农村居民人均纯收入由4006元增加到8020元，比2005年翻1番。就业规模不断扩大，社会保障体系逐步完善，文化、教育、医疗等社会事业加快发展。居住条件明显改善，城市人均住房使用面积达22.1平方米，农村住房砖瓦化率达87.6%，累计改造棚户区1089万平方米、农村泥草房1151万平方米，建设经济适用住房896.7万平方米，新增廉租房受益户13998户，为6732户家庭发放经济适用住房货币补贴2.2亿元。成功举办第24届世界大学生冬季运动会。荣获“全国双拥模范城”6连冠。

　　各位代表，2010年是“十一五”时期的收官之年，也是我市全面实施“北跃、南拓、中兴、强县”发展战略的第一年。市政府和全市人民一道，紧紧围绕“超越自我、再塑形象、奋起追赶、努力晋位，把哈尔滨建设成为现代大都市”的奋斗目标，推动经济社会发展取得了可喜成果。

　　一是新战略实施开局良好。支撑新战略实施的500个重点项目取得明显进展，完成投资1092.8亿元。“北跃”战略推进迅速，松浦大桥提前半年竣工通车；科技创新城创新园和产业园启动基础设施建设，39个研发和产业项目开工建设；“北国水城”骨干工程量完成14.9%，我市被国家确定为水生态系统保护与修复试点城市。“南拓”战略全面展开，哈南工业新城投入建设资金80亿元，新开发面积20平方公里，209个工业项目开（复）工。“中兴”战略成果显著，新建改造长江路、铁路街、友谊西路等道路202条，建设文昌高架桥、征仪路跨线桥等各类桥涵57座，维修道路500条（段），城市道路完好率达90%。“强县”战略逐步显效，县域经济总量突破1000亿元，县域规模以上工业增加值达130.6亿元。宾西经济技术开发区晋升国家级开发区。

　　二是三次产业协调发展。旅游、文化、物流、金融等现代服务业加快发展，旅游总收入突破380亿元，增长23%；哈尔滨文化中心、音乐厅、群力文化产业示范基地等项目规划建设取得进展；龙运物流园区、哈药集团物流配送中心等重大物流项目建设取得成效；美国摩根大通和香港东亚银行在我市设立分支机构获银监会批筹，哈尔滨银行在6省（市）设立分支机构，九洲电气等3户企业成功上市，结束了我省7年没有企业在内地上市的历史。规模以上工业增加值实现732.1亿元，增长17.2%；经济效益综合指数达199.8，提高6.2个百分点。克服春涝、低温冷害等多种自然灾害，粮食总产量达251.8亿斤，创历史新高；实现畜牧业产值364.2亿元，增长6.5%，占农林牧渔业总产值46.4%。

　　三是大项目建设提速增效。积极开展“项目建设提速年”活动，项目投资规模、层次质量、建设速度前所未有。完成全社会固定资产投资2651.9亿元，增长40.2%，其中亿元以上项目完成投资1126.6亿元，占全市投资比重达42.5%。大项目建设保障力度加大，市级以上重点项目审批全部纳入“绿色通道”；争取项目建设用地24.15平方公里，接近“十一五”时期前4年的总和；与各类金融机构签署授信额度1155亿元，“哈投”、“城投”、“水投”、“哈西”等融资平台落实贷款合同422亿元，实际到位资金208亿元；项目招商引资规模扩大，全年实际利用外资7亿美元，国内经济技术合作资金到位额517.2亿元，分别增长15.7%和40%。

　　四是城市管理水平明显提升。针对城市管理中存在的突出问题，集中打好环境整治“三大战役”，完成裸土地面硬化、绿化铺装513万平方米，新植树木102万株，新增绿地800公顷，绿化覆盖率达38%；拆除违章建筑8万多平方米，清理各类广告牌匾9700余块，改造旧有居民庭院2640个，城市环境卫生和市容面貌明显改观。依法严格治理交通环境，在路桥等基础设施大规模建设时期基本保障了道路畅通。大力推行网格化管理模式，实施清扫保洁分级管理，建立垃圾收运新体系，城市管理效率明显提升。加大环卫基础设施投入，市区两级财政投入1.76亿元，购置清扫保洁设备617台（套），城市道路机械化清扫率和水冲洗作业率均达到20%以上。全面启动“三沟一河”综合治理工程，清运沿河垃圾12万吨，取缔污染企业14户，铺设截污管线43.9公里，“三沟”污水全部实现截流，“三沟一河”环境卫生纳入城市一体化管理。

　　五是社会事业全面进步。各级各类教育均衡发展，“两基”目标顺利实现；投入近2亿元，完成部分学校危房校舍加固改造；150所义务教育学校达到标准化，义务教育阶段民办公助学校改制全部完成。医药卫生体制改革不断深化，城市社区卫生服务覆盖率达98％，新农合参合率达99.1%，106个基层医疗卫生单位实行药品零差率销售。人口自然增长率控制在4.5％。以内。文化、体育事业蓬勃发展，社区图书馆、乡镇文化站、“农家书屋”等贴近群众、服务群众的文化设施进一步完善，成功举办第30届“哈尔滨之夏音乐会”，我市被联合国授予“音乐之城”称号；安装健身路径1300件（套），经常性参加体育锻炼人数占总人口的33%。扎实推进“平安哈尔滨”创建活动，刑事案件万人发案率在全国副省级城市中保持最低；重特大安全事故得到有效遏制；实行信访联合接待新机制，信访接待大厅投入使用，信访秩序有所好转。民族宗教、国防和预备役等工作进一步加强，残疾人、妇女儿童等事业取得新进展。

　　六是精神文明和民主法制建设得到加强。“三优”文明城市和全国文明城市创建工作扎实推进，和谐社区建设、公民道德主题实践活动、群众性精神文明基础创建活动成效明显，公民文明素养、道德水平进一步提高。依法行政能力不断增强，自觉接受人大依法监督和政协民主监督，人大代表建议、政协提案办复率100%。提请市人大常委会审议地方性法规草案4件，制定政府规章16件。发展环境得到改善，实行改善经济发展环境21条措施，成立非公有制企业维权投诉服务中心，开展12个纠风专项治理行动，取消审批前置要件265个，收费项目减少到83项。

　　七是人民群众生活水平有新提高。年初承诺的60项惠民行动项目超计划完成，总投入达317.8亿元，是上年的近2倍。就业渠道进一步拓宽，城镇新增就业10.8万人，转移农村劳动力131万人。棚户区和泥草房改造力度加大，启动棚改拆迁项目42个，拆迁面积518万平方米，涉及拆迁户3.3万户，竣工318万平方米，回迁居民1万户；改造农村泥草房4.3万户，我市成为国家首批利用住房公积金贷款支持保障性住房建设试点城市。社会保险覆盖面持续扩大，“五七工”、“家属工”参保工作全面完成，新农保试点工作扎实推进，养老、失业、医疗保险覆盖面分别达91.2%、95.5%和94.5%。城市和农村低保标准进一步提高，社会救助范围不断扩大。城市每千名老人拥有养老床位20张，农村“五保”对象集中供养能力提高到60%。

　　各位代表，“十一五”时期取得的辉煌成就来之不易，特别是在市委确定的新战略引领下，2010年工作积累的实践经验弥足珍贵，创造的精神财富影响深远，为哈尔滨“十二五”乃至今后一个时期跨入一条既符合时代要求、又符合市情实际的科学发展道路，奠定了坚实基础。在此，我代表市人民政府，向全市各族人民，向各民主党派、工商联、无党派和社会各界人士，向驻哈部队、武警官兵和中省直驻哈企事业单位，向关心、支持哈尔滨建设和发展的港澳台同胞、海外侨胞和国际友人，致以崇高的敬意和衷心的感谢！

　　在肯定成绩的同时，我们也清醒地认识到，我市经济社会发展还存在许多矛盾和问题。一是经济总量小，发展速度相对缓慢，经济综合实力和核心竞争力相对较弱问题仍然突出。二是产业结构不尽合理，农业基础薄弱，农业现代化任重道远；传统产业仍占主导地位，战略性新兴产业比重不大、规模小；服务业比重虚高，现代服务业发展滞后，加快经济结构战略性调整、促进经济发展方式转变已经刻不容缓。三是城乡二元结构矛盾突出，经济社会发展不均衡，县域经济实力比较弱，工业化、城镇化水平偏低，逐步缩小城乡差距、实现城乡统筹一体化发展仍然是需要着力破解的难题。四是体制性、机制性障碍没有从根本上消除，对外开放的层次和水平不高，改革创新的任务仍然十分艰巨。五是城市基础设施历史欠账较多，地下管网设施老化问题比较严重，停水、停热事故时有发生；路网功能改造提升任务艰巨；环境综合整治有待进一步深化，老旧居民小区物业“失管失养”问题比较突出，城市管理长效机制尚需完善。六是经济发展的资源环境约束日趋强化，节能减排、污染治理、环境保护压力增大，迫切需要加快构建资源节约、环境友好的生产方式和消费模式，增强经济社会可持续发展能力。七是一些涉及人民群众切身利益的民生问题还没有解决好，基本公共服务体系不够完善，与让人民群众生活得更方便、更满意、更幸福的要求相比还有不小差距；城乡居民收入水平不高，低收入困难群众比较多，出行难、看病难、保障性住房供给不足等问题不同程度存在。八是有些部门公务人员服务意识不强、工作作风不实、办事效率不高，对一些事关群众切身利益和企业发展的公共事务漠不关心、推诿扯皮；腐败问题时有发生，在一些领域、一些地方还比较严重。我们将高度重视这些问题，切实采取有效措施，认真加以解决。

　　二、“十二五”时期的主要目标和战略重点

　　“十二五”时期是我市深入实施新战略、为全面建成小康社会奠定坚实基础的关键时期。在这个历史发展阶段，我们仍然处于我国发展可以大有作为的重要战略机遇期，机遇挑战并存，机遇大于挑战。我们既要珍惜机遇、抓住机遇、用好机遇，又要正视挑战、应对挑战、战胜挑战，主动适应宏观环境变化，牢牢把握科学发展规律，有效化解各种社会矛盾，努力增进全市人民福祉，以更加超越自我的精神状态和更加奋发有为的务实作风，推动哈尔滨经济社会更好更快更大发展。今后5年我们努力奋斗的目标是：

　　———民生状况明显改善。坚持把发展为了民生摆在首位，切实让人民群众得到更多实惠：一是城乡居民收入普遍较快增加。预期到2015年，实现城乡居民收入增长和经济发展同步，城市居民人均可支配收入、农村居民人均纯收入、中低收入群体收入和城乡居民最低生活保障标准年均增速均达到12%以上，最低工资标准年均增长15%左右，力争城乡居民收入水平在同等城市中实现晋位。二是住房保障水平大幅提高。力争解决13万户低收入和中等偏下收入家庭住房困难，中低收入群体人均住房面积不低于15平方米，城区四环路内棚户区改造全部完成，棚户区居民全部入住新居，农村泥草房改造基本完成。三是就业规模持续扩大。多渠道开发就业岗位，提高劳动者就业能力，促进充分就业，新增城镇就业55万人，城镇登记失业率控制在4.5%以内。全力实施百万农民工技能培训工程，增加劳务收入100亿元。未来5年，将成为我市改革发展成果惠民力度最大的5年，成为全市人民生活明显改善的5年，成为人民群众生活更方便、更满意、更幸福的5年。

　　———产业核心竞争力显著增强。坚持把结构调整作为加快转变经济发展方式的主攻方向，着力实施产业“倍增计划”，预期到2015年，装备制造、食品、医药、石化4大优势产业增加值实现5年翻番，重点培育装备制造、食品2个销售收入超千亿元产业。民用航空、生物、新材料、新能源装备、电子信息、绿色食品6大战略性新兴产业增加值实现4年翻番，重点打造新能源装备、电子信息和绿色食品3个超百亿元产业。金融、物流、文化、信息服务4大产业增加值实现4年翻番，旅游总收入突破960亿元，占生产总值比重达15%。

　　———基本公共服务体系逐步完善。坚持统筹发展社会事业，注重加强社会建设，加快建立健全符合市情、比较完整、覆盖城乡、可持续的基本公共服务体系。我们构建的基本公共服务体系，既要符合我市经济发展水平，又要与人民群众不断增长的需求相适应，涉及基本民生保障的各个主要领域，既覆盖城镇居民，也覆盖农村居民，特别是社会养老保险、基本医疗保险和社会救助要实现城乡全覆盖，农村社区、城镇社区便民医疗、文化、体育设施实现广覆盖，推进基本公共服务均等化。学前3年教育毛入园率提高到75%，城区和县镇义务教育学校全部达到标准化，职业教育取得突破性进展。12所县级医院全部建成2级甲等以上标准医院。新增城市道路130公里，道路完好率达95%，全面改造供水、排水、供气、供热等老旧地下管网，根本扭转城市基础设施落后状况。公交优先发展战略取得实质进展，公交在城市交通总出行中的比重提高到45%以上。

　　努力实现上述目标，最大限度地满足全市人民的新期待，要求我们切实做到以下几点：

　　（一）必须准确把握科学发展总体思路。深入贯彻落实党的十七届五中全会、省委十届十四次全会和市委十二届十次全会精神，坚持以科学发展为主题，以加快转变经济发展方式为主线，以改革开放和科技创新为动力，以改善民生和维护社会稳定为根本，紧紧围绕北国水城、工业大城、科技新城、文化名城、商贸都城的发展定位，全力实施“北跃、南拓、中兴、强县”发展战略，推动经济社会更好更快更大发展，为把哈尔滨建设成为现代大都市打下坚实基础。

　　（二）必须持之以恒深入实施新战略。按照“以松花江为纲———一江居中、两岸繁荣”的战略构想，持续推动城市发展空间向北跨越、向南拓展、向县域延伸。

　　一是“北国水城”和科技创新城建设取得明显进展。“北国水城”中“2纵”、“2横”、14个湖泊基本建成，32座闸站、79座桥梁及相关附属景观设施投入使用，江北水系呈现玉带倩影，水生态功能得到恢复和保护。科技创新城起步区全部建成，交通、教育、医疗、居住等配套功能基本完备，新材料、新能源、生物医药、光机电一体化、电子信息等5大产业集群作用凸显，成为全省高端创新人才集聚区、高端技术研发集成核心区、高端成果孵化运用主导区。联通松花江两岸的跨江通道达到13条，沿江建设的哈尔滨大剧院投入使用，文化中心成为新地标，金融中心功能作用初步显现。

　　二是哈南工业新城产业格局基本形成。力争开发建设85平方公里，形成辐射贯通周边各工业园区的战略格局，航空、汽车等优势产业竞争力明显增强，云计算、高端装备制造等战略性新兴产业集聚发展，工业总产值突破3500亿元，成为全省最大的现代产业聚集区和全国重要的新型工业化示范区。

　　三是中心城区城市功能显著提升。老城区改造和哈西、群力新区开发提速，影响群众生产生活的市容卫生、公共交通、生态环境、基础设施等问题得到有效解决，中心城区承载功能、生态质量、容貌形象、居住条件、管理水平和辐射带动能力明显提升，再现欧陆风情的独特魅力。大力发展都市型工业、总部经济和现代服务业，城区经济进一步繁荣。

　　四是县域经济社会统筹协调发展。实施区域发展和主体功能区战略，推动城乡形成区域互动、优势互补、相互促进、共同发展新格局。充分发挥各级各类开发区和工业园区的示范带动作用，因地制宜，突出特色，错位发展。以水利为重点的农业基础建设明显加强，农田水利化程度达到70%，综合机械化程度达到94%，畜牧业占农林牧渔业比重达到50%，粮食产能稳定在250亿斤以上，农业生产规模化、作业机械化、经营产业化、产品标准化水平显著提高。新农村建设取得重大进展，建成一批特色突出、设施良好、功能完备、环境优美的小城镇。加大户籍制度改革力度，制定完善相关配套政策，力争把符合条件的百万农业转移人口逐步转为城镇居民，城镇化水平提高到65%。基本实现城乡规划、产业发展、基础设施、公共服务、市场和管理一体化，推动城乡走上生产发展、生态良好、生活富裕的文明发展道路。

　　（三）必须切实加快转变经济发展方式。坚持在发展中促转变、在转变中谋发展，努力提高发展的全面性、协调性、可持续性。

　　一是结构调整取得实质性进展。按照稳农业、强工业、提档升级第三产业的思路，加快形成以服务经济为主的现代产业体系。预期到2015年，三次产业结构调整到8:40:52。

　　二是科技进步和创新支撑作用明显增强。科技进步对经济增长的贡献率提高到60%以上，全社会研究和试验发展经费支出占地区生产总值的比重达到2.5%以上，高新技术产业产值年均增长20%以上，基本建成创新特色鲜明、创新活力充沛、创新氛围浓厚的国家创新型城市。

　　三是资源节约利用和环境保护得到加强。大力发展循环经济，推广低碳技术，万元GDP综合能耗累计下降16.5%，城市生活污水、垃圾和县（市）生活垃圾无害化处理率分别达到95%、85%和72%以上，水、气主要污染物排放累计下降15%和5%，城市集中供热普及率达到85%，市区环境空气质量2级及2级以上天数达到325天，消除内河劣5类水质，松花江出境水质达到3类标准。森林覆盖率达到46%。

　　通过创新驱动、转型发展，哈尔滨经济综合实力显著增强，预期全市地区生产总值年均增长12%以上，人均地区生产总值突破1万美元，地方财政一般预算收入实现翻番，全社会固定资产投资年均增长30%。全国文明城市、国家环保模范城市、卫生城市和园林城市创建活动取得实质进展，城乡载体功能、服务功能和综合保障功能明显提升，全省经济发展支柱、科技创新先导、城乡建设表率、服务全省平台的龙头带动作用更加突出。

　　（四）必须坚定不移深化改革开放。关系经济社会发展全局的重大改革向纵深领域推进，更具活力、更富效率、更加开放的体制机制逐步形成。

　　一是深化经济管理体制改革。毫不动摇地鼓励、支持、引导非公有制经济发展，努力营造各种所有制经济依法平等使用生产要素、公平参与市场竞争、同等受到法律保护的制度环境，预期到2015年，非公有制经济比重增加到60%以上。积极推进国有经济战略性调整，深化国有企业改革，完善现代企业制度，促进国有资本向城市基础设施、战略性新兴产业等领域集中。

　　二是深化财税金融体制改革。推进投融资体制改革，加快发展创业投资、工业投资、城建投资等大型国有投融资平台，大力发展上市公司。进一步理顺各级政府间财政分配关系，增加一般性转移支付规模和比例，加强县级政府提供基本公共服务财力保障。

　　三是深化农村综合改革。加快农村产权要素市场化、资本化进程，新型农业服务体系实现区县（市）全覆盖，土地承包和流转制度进一步完善，农民组织化程度明显提高，土地适度规模经营面积稳步扩大，水利、林权、农村金融等改革取得积极进展。

　　四是深化行政管理和社会管理体制改革。加快转变政府职能，推进政事企分开，依法调整政府、市场、企业之间的关系，减少政府对微观经济活动的干预。推进事业单位改革，建立有效的用人机制和考核分配激励机制。加大社会体制改革力度，力争在医药卫生体制、教育体制、文化体制改革等方面取得实质进展。

　　五是深入实施互利共赢开放战略。以建设中俄友好合作示范城市为目标，深度参与东北亚区域合作，积极打造服务全国的对俄合作交流平台，推动对俄合作战略升级。进一步优化对外贸易结构，促进出口结构转型升级，积极扩大进口，更好地发挥哈尔滨内陆港国际联运优势，推动对外贸易平衡发展。预期到2015年，市属外贸进出口总额年均增长15%。进一步加大招商引资力度，加强区域经济技术合作，实际利用外资年均增长10%以上，国内经济技术合作资金到位额年均增长20%以上。

　　三、2011年主要任务

　　2011年是中国共产党成立90周年，是“十二五”规划开局起步之年，做好今年工作具有十分重要的意义。按照市委十二届十次全会的要求和部署，我们将凝聚全市力量，集中开展“招商引资项目落实年”和“政风建设年”活动，全力以赴抓大事、干实事，千方百计促发展、惠民生。

　　在保障和改善民生方面，深入实施惠民行动，涵盖8个方面、40个项目，计划投资达205亿元。城市居民人均可支配收入和农村居民人均纯收入分别增长12%。城镇新增就业9.4万人，登记失业率控制在4.5%以内；转移农村劳动力134万人。新建各类保障性住房4.2万套，拆迁改造棚户区360万平方米，改造农村泥草房4万户。新增标准化义务教育学校80所。6个县（市）人民医院达到2级甲等以上综合医院标准。政府举办基层医疗卫生机构全部实行基本药物制度和基本药物零差率销售。

　　在经济社会发展方面，注重实现经济发展速度和结构质量效益相统一，经济发展与人口资源环境相协调。预期地区生产总值增长12%以上。地方财政一般预算收入增长15%以上；社会消费品零售总额增长15%；人口自然增长率控制在5.8％。以内；主要污染物排放量削减率达到“十二五”总任务的15%。新上一批支撑新战略实施、促进结构调整的大项目，全年市级以上重点项目达到300个，年度投资超过800亿元，带动全社会固定资产投资增长30%。

　　在城乡建设和管理方面，进一步畅通南北跨江交通，确保阳明滩大桥、松花江公路大桥扩建工程年底竣工通车。不断完善城乡路网体系，全年新建续建城区路桥工程130项，维修道路330条（段）；建设农村公路500公里，竣工通车300公里。深入推进“三沟一河”综合整治，何家沟、马家沟郊区段和阿什河城区段建设任务全面完成，阿什河水质基本消除劣5类。

　　我们将统筹抓好以下10项工作：

　　（一）着力调整优化结构，切实增强工业经济发展实力。工业是哈尔滨的立市之基、强市之本。没有工业的振兴，就不会实现老工业基地的全面振兴。我们将牢牢把握新型工业化发展方向，着力调整优化工业结构，不断提升产业集中度和竞争力，确保规模以上工业增加值增长14%以上。

　　一是调整优化工业结构。做大做强4大优势产业，增加值占规模以上工业保持80%以上。落实6大战略性新兴产业发展规划，重点实施核电主泵电机、新能源汽车、民用航空基地等一批重点项目和示范工程。继续抓好能源装备、汽车、精细化工等8大产业链，着力实施汽车自动变速器等一批产业链节点项目。积极培育主业突出、带动力强的大企业、大集团，重点支持哈飞、哈药、轻型车厂等企业扩大国内外合作。继续实施品牌战略，加快国家商标战略示范城市建设，提高企业产品市场竞争力。围绕工业化与信息化融合，组织开展装备制造业“两化”融合指标体系试点，重点启动哈电集团协同制造平台等项目建设，提升工业信息化水平。

　　二是壮大工业园区规模。重点推进哈南工业新城开发建设，确保新哈缆等100个项目开工建设，云计算中心等100个项目签约入驻，启动京哈公路互通立交枢纽、哈五路拓宽改造等基础设施配套工程，完成新开发面积20平方公里。加快宾西经济技术开发区建设，启动路网改造、蜚克图河流域综合治理等重点工程，突出抓好哈工大奥瑞德、天路新能源等一批大项目建设，新增入区项目30个，完成投资40亿元。抢抓省在哈大齐工业走廊再建食品、生物、化工、装备制造、物流等五个大园区机遇，以呼兰河工业核心示范区为重点，加快哈大齐工业走廊哈尔滨段建设，新增入区项目100个，完成投资180亿元。整合优化园区资源，形成产值超50亿元工业园区6个，新增园区工业总产值400亿元。

　　三是突出抓好重大工业项目。结合开展“招商引资项目落实年”活动，加快建立完善市、区、县（市）领导包保责任体系和具体推进标准、考核办法，形成“一个项目、一个班子、一套人马”推进机制，力促大项目早谋划、早开工、早落实。重点抓好100个亿元以上大项目，确保50个重点项目竣工投产，力争工业投资增长25%以上。全年新谋划3000万元以上项目100个。

　　（二）着力夯实农业基础，切实提高农业和农村经济发展水平。我市大城市、大农村特征鲜明，破解“三农”问题任务十分繁重。我们将按照城乡统筹发展的要求，全面贯彻工业反哺农业、城市支持农村和多予、少取、放活的方针，积极推进传统农业向现代农业转变，加大强农惠农力度，加快农业现代化、县域工业化、农村城镇化进程，确保农林牧渔业总产值增长5.5%，县域生产总值和财政收入分别增长15%和20%。

　　一是进一步加强农业基础建设。围绕实施增产50亿斤粮食产能工程，加快建设高标准良田、改造中低产田，加强气象灾害预报预警，提高粮食综合生产能力。加强灌区配套、节水改造和提水泵站建设，完成堤防险工弱段和小型病险水库除险加固任务，推进少陵河、巴兰河等中小河流治理。加大农机补贴力度，新增大型农机具400台（套），农机田间作业综合机械化率达82%。加快农机装备产业基地建设，力争主营业务收入超过15亿元,积极推进福田雷沃、约翰·迪尔等重点企业尽快落户我市发展。

　　二是大力推动农业结构调整。重点调优种植业，做大畜牧业。积极扩大高产、优质、高效作物面积，切实提高优良品种覆盖率。全力实施“提水扩稻”工程，新增水田面积75万亩。大力发展循环农业、设施农业和旅游观光农业，支持省农业科技示范园建设，建成市现代循环农业示范园。加快蔬菜基地和有机农产品基地建设，棚室蔬菜生产面积达到17万亩，绿色有机食品认证面积扩大到800万亩。加快发展现代畜牧业，新建规模化、标准化畜牧场和养殖园区50个。强化农产品质量监管和重大动物疫病防控，确保农产品质量安全。

　　三是切实增强县域工业发展实力。以做专做强县域工业园区和农产品加工企业为重点，努力提高县域工业化水平。继续扶持壮大产业化龙头企业，新增规模以上企业5家，产业化基地面积扩大到1797万亩。进一步做大拉长水稻、生猪、乳品等10大产业链，打造农业名优品牌，提高农产品附加值，力争县域工业增加值增长15%以上。

　　四是积极稳妥推进城镇化。坚持走“强县先强镇”的路子，实施“百镇提档升级、20镇试点引领、6镇示范样板工程”，率先在基础较好的区域建设一批有产业支撑、各具特色的小城镇。研究制定促进符合条件的农民转变为城镇居民的政策措施，不断提高城镇化水平。启动第三批市级新农村试点，推进平新镇、红旗乡等一批村庄资源整合，建设新型居民集中区，完成饮水安全工程224处，高标准绿化村屯100个。抓好城乡建设用地“增减挂钩”试点，盘活农村建设用地资源。切实解决好失地农民住房、就业、社保、医疗、教育等问题。完成第五批116个村扶贫开发整村推进任务。

　　（三）着力发展现代服务业，切实提高服务业层次和质量。服务业具有广阔的市场空间和巨大的需求潜力，对拉动产业发展、扩大就业、带动消费优势明显。我们将抢抓国家服务业综合改革试点区域政策机遇，大力发展现代服务业、生产性服务业和生活性服务业，提高服务业增长的质量、效益和水平，确保第三产业增加值增长12.5%以上。

　　一是做大做强旅游业。以打造国际旅游城市为目标，进一步提升“冰城夏都”旅游品牌形象，全力推进北国风光特色旅游开发区建设，力争全年旅游收入增长20%。以保护开发“万顷松江湿地、百里生态长廊”为核心，深度挖掘整合冰雪、湿地、森林、金源文化等资源优势，促进旅游业成为助推经济发展、调整经济结构、改善生态环境、增加居民收入的重要支撑。启动呼兰三家子旅游小镇、长岭湖旅游风景小镇等旅游名镇规划和具体项目推进工作，引导发展乡村旅游。加快推进金河湾湿地公园等湿地游项目，着力打造5条松花江湿地游黄金线路，加大宣传推介力度，办好首届“中国哈尔滨松花江湿地旅游文化节”，切实增强湿地旅游的影响力和吸引力，努力把湿地旅游逐步打造成继冰雪旅游之后的又一国际品牌。

　　二是加快培育壮大物流和信息服务业。按照物流产业布局，加快推进龙运、新香坊等物流园区项目建设，规划建设重型装备及机电产品物流中心、哈药集团物流中心、化工物流配送中心、绿色食品博览中心等专业物流中心和配送中心，积极谋划哈南工业新城国际物流港、江北综合物流产业园、果蔬净菜物流园等项目。加快打造哈满、哈绥、哈大、哈黑、哈同等陆路和航空物流大通道。扎实推进“三网融合”试点，鼓励广电、电信业务相互进入、融合发展。突出抓好国际数据城、物联网大厦、电子商务、电子政务等重大应用工程，大力推广物联网技术在产品溯源、环境保护、危险品储运等领域应用，促进汽车传感器、光纤传感器、电力电子等产业加快发展。

　　三是大力发展文化产业。抓好平房动漫园、群力文化产业园、太阳岛文化产业园、出版印刷产业园、哈师大现代文艺产业园和呼兰黑龙江广播影视产业园6大文化产业园区建设，重点推进哈尔滨大剧院、职工文化艺术宫、博物馆、美术馆、音乐厅等一批标志性城市文化设施建设，积极打造中华巴洛克等特色文化街区。抓住国家第二批文化体制改革试点机遇，进一步深化电影公司、文艺演出院团等经营性文化单位转企改制，成立哈尔滨演艺集团，推动广播电视、新闻出版等媒体转型。

　　四是积极发展金融业和商贸流通业。改善金融生态环境，积极引进境内外金融机构，支持哈尔滨银行做优做强，加快松北金融中心等重点项目建设。优化城市商圈布局，推进老城区商圈提档升级，规划建设松北、哈南、群力、哈西新商圈。深入实施“万村千乡市场工程”。积极培育和发展信息、咨询、会展、创意等新兴服务业。

　　五是鼓励引导发展生活性服务业。坚持社会化发展方向，将生活性服务业作为提供更多就业岗位、方便人民群众生活、培育新经济增长点的重要服务产业。鼓励扶持社会力量开办家政服务、养老服务、患病陪护、家庭教育和家庭用品配送等生活性服务机构，建立健全多种形式的生活性服务体系，满足城乡居民多样化的服务需求。全年新增养老床位1000张，千名老人拥有养老床位达到22张。

　　（四）着力推进改革开放，切实增强经济发展的动力和活力。实现我市经济社会更好更快更大发展，迫切需要不断完善有利于科学发展的体制条件和开放环境。我们将以开展“招商引资项目落实年”活动为载体，进一步深化改革、扩大开放，最大限度地增强发展动力、释放发展活力。

　　一是努力扩大招商引资和对外贸易。落实招商项目计划方案，成立招商项目推进组，大力开展普遍招商、重点招商、专项招商、委托招商，预期全年实际利用外资增长10%，国内经济技术合作资金到位额增长30%。发挥政策性资金杠杆作用，灵活运用贷款贴息、贷款担保、定额补助等政策，吸引更多的民间资本和外来资金参与重大项目建设。高水平办好第22届“哈洽会”。继续巩固传统市场，积极抢占新兴市场，调整优化出口商品结构，重点扩大哈飞汽车、光宇蓄电池等地产名牌产品出口。做大做强对俄出口加工基地，吸引一批境外、省外企业来我市兴办对俄出口加工企业，新增对俄贸易超千万美元企业5至10户，逐步把哈尔滨打造成为哈牡绥东对俄贸易加工区的龙头。继续抓好国家服务外包示范城市建设。预期市属外贸进出口总额增长15%。

　　二是深入推进重点领域和关键环节改革。加快以产权制度改革为核心的国有企业改制重组，完善法人治理结构，重点推进哈电缆厂、哈一工具厂合资合作，哈轴集团、电碳厂整体搬迁、退城进郊。继续抓好厂办大集体企业改革。深化财政管理体制、投融资体制改革，完善参与式预算、财政支出绩效评价等管理制度，提高预算编制和执行的科学化水平；清理规范政府投融资平台，增强市场融资能力。深入推进农村产权制度和集体林权制度改革，基本完成登记确权颁证任务。建立农村产权交易流转平台，鼓励农民采取多种方式流转土地承包经营权，规模经营面积扩大到350万亩。健全农业社会化服务体系，新增农民专业合作社400个。深化行政管理体制改革、事业单位分类改革和公用公益事业改革，逐步解决好政事企不分等问题。

　　三是大力发展非公有制经济。放宽市场准入，扩大民间投资，支持骨干企业、民营资本参与国企改革，进入基础产业、基础设施建设、市政公用事业、社会事业、金融服务和商贸流通等领域。建立健全创业辅导、融资担保、人才培训等服务体系。深入实施中小企业创业成长工程，全年新增收入超亿元企业10户以上，规模企业50户以上。

　　（五）着力提高自主创新能力，切实增强科技进步对经济社会发展的驱动作用。依靠科技创新驱动经济社会更好更快更大发展，是我市的比较优势和战略选择。我们将抢抓国家创新型试点城市机遇，把技术进步与产业结构优化升级、改善民生紧密结合起来，着力在重点领域取得突破，科技进步对经济增长的贡献率提高到53%。

　　一是加快科技创新城建设。全力推进创新园起步区和产业园起步区征地拆迁及基础设施建设，完成投资35亿元，累计开发面积达9.34平方公里。加快省新兴产业技术创新服务平台、国际农业科技创新中心等标志性项目建设，启动兵装集团热能装备基地、LED产业园区等一批产业项目，确保中科院哈尔滨产业技术创新与育成中心、国际科技合作基地、软件园建成并投入使用。不断提升高端研发项目吸引力和集聚度，新引进项目开工建设面积160万平方米以上。

　　二是增强科技创新能力。重点实施科技创新引领等“6大创新工程”和100项重点科技计划，加快铝镁合金新材料等6个国家级高新技术产业基地建设。加大引进消化吸收再创新力度，重点抓好“哈科会”签约项目合作研发及产业化。依托大学、大所、大厂科技资源优势，完善产学研合作机制，壮大产业技术联合体，促进新能源汽车等7条高新技术产业链发展，提升科技成果本地转化水平，实现高新技术产业产值增长20%以上。

　　三是健全科技创新服务体系。继续推进技术研发、科技资源信息共享、科技企业孵化、技术转移服务和科技投融资5大创新平台建设。抓好国家知识产权工作示范城市建设，实现专利申请量增长10%以上。实施“人才强市”战略，完善项目建设人才服务“绿色通道”，引进和用好高层次人才，统筹抓好以领军人才和高技能人才为重点的各类人才队伍建设。

　　（六）着力加强城乡规划建设管理，切实增强城乡载体和服务功能。城乡规划建设管理事关发展、惠及民生，倍受期待和瞩目。我们将以深入实施“中兴”战略为牵动，统筹推进城乡建设，加快实现一体化步伐。

　　一是强化规划的统领作用。围绕实施新战略，落实国务院批准的新一轮城市总体规划，修编控制性详细规划，完善万顷松江湿地保护与利用规划，编制招商引资及可收储土地布局规划。高标准搞好重点地区、重要地段城市设计。启动建设哈尔滨市城乡规划展馆。加强城市基础地理信息数据库建设，提升城乡规划科技应用水平。依法严格执行城乡规划，切实维护规划的权威性和严肃性。

　　二是加快推进以路桥为重点的城乡基础设施建设。城市东部地区重点抓好东巨路、香福路等道路改造建设，为保护开发松江湿地、促进东部地区加快发展创造有利条件。西部地区重点启动实施哈平路、哈双南路等道路新建改建，支撑群力新区和哈西地区联通发展，密切哈南工业新城与城市中心区的交通联系。

全面完成老机场路二期改造任务。中部地区重点结合哈站改造，配套实施哈站南广场改造、北广场新建和区域交通路网改造等关联工程，从根本上解决哈站周边区域交通拥堵问题。加快宣化街高架桥建设，确保“哈洽会”前建成通车。继续推进地铁一号线建设。积极配合铁道部如期完成哈西客站建设任务。加大出城口路桥改造力度，续建、新建哈阿公路等10条城市出口路和7个高速公路互通立交出口，同步推进江南中环路改建。启动双琦生活垃圾综合处理厂建设，推进城市餐厨垃圾处理厂、粪便处理厂建设。完成依七高速依兰段、哈肇公路依兰段、铁通公路尚志至五常段、哈五公路平房至拉林段改造任务。

　　三是加大城市管理力度。深入开展市容卫生环境综合整治，全面实行环卫清扫保洁和清冰雪分级管理，完善垃圾收运机制，增加环卫设施，打造干净城市。加强牌匾广告、违章建筑、违规占道等综合治理，推进铁路环线及哈同入城口周边环境综合整治，高标准打造道外沿江风景线。强化交通环境整治，完善市区交通流量调控措施和配套交通设施，新增停车泊位1万个，提高交通管理水平。启动数字城管扩建工程，增加视频监控点位，建立城市地理信息查询系统，不断提高城市管理精细化、网格化、智能化水平。坚持市、区（县市）、街道3级管理体制，强化区、街城市管理职能，完善建管交接机制，切实发挥街道办事处推动城市管理、组织公共服务、指导社区建设、促进社会和谐的重要作用。

　　（七）着力推进资源节约型、环境友好型社会建设，切实改善城乡生态环境。节约资源和保护环境是实现可持续发展的必然要求，也是加快转变经济发展方式的重要着力点。我们将立足当前、兼顾长远，坚持不懈抓好资源节约和环境保护，不断增强可持续发展能力。

　　一是加强水生态保护与修复。实施水生态保护与修复总体规划，构建一体化城市水网体系。围绕“2纵、4横、18湖”水系规划目标，加快“北国水城”建设，重点抓好松北灌排体系改扩建，开挖渠道23.8公里，完成4个湖泊及部分设施建设，力争实现通水见绿。实施松花江北岸东方红、三家子、松浦、外贸堤防升级达标和堤顶道路工程建设，力争实现区间道路通车。

　　二是加强节能减排。抓好重点领域和重点企业节能降耗，推行合同能源管理，开展重金属、产能过剩行业清洁生产强制审核，严格依法淘汰落后产能。加快推进城镇污水收集管网建设，完成阿城、双城污水处理厂增容工程，城市生活污水处理率提高到80%。加快电镀生态园和哈三电厂脱硫项目建设，全面推行排污在线监控和二氧化硫排污权交易制度，依法从严查处非法排放企业。

　　三是改善城乡环境质量。实施磨盘山水源地保护规划，推进城镇集中式饮用水源治理工程，保障城乡供水安全。继续推进哈热电厂六期、国电平南热电厂新建、道里集中供热三期扩建等项目建设，加快拆炉并网进度，新增集中供热面积500万平方米。加快公交车双燃料改造，强化机动车尾气和施工扬尘污染防治，确保市区环境空气质量2级及2级以上天数不低于315天。实施城乡大绿化工程，农村造林10万亩，森林覆盖率达45.5%；城区新植树木70万株，新增绿地400公顷，绿化覆盖率达38.9%。深入推进农村环境综合整治，加快村屯污水、垃圾、规模化养殖污染治理项目建设，新建示范乡镇、村屯36个。治理水土流失面积36万亩。

　　（八）着力解决事关人民群众切身利益的实际问题，切实保障和改善民生。保障和改善民生是加快转变经济发展方式的根本出发点和落脚点，也是不断解决人民群众最关心、最直接、最现实利益问题的必然要求。我们将以让人民群众生活得更方便、更满意、更幸福为目标，坚持发展为民生，扩大内需保民生，加快转变促民生，切实提高人民群众生活质量和水平。

　　一是全力解决好人民群众反映最强烈、要求最迫切的实际问题。第一，针对物业管理不规范、不到位、居民与物业企业矛盾突出等问题，严格执行法律、法规，完善相应政策措施，发挥属地管辖优势，切实提高物业管理和服务水平。第二，重点整治老旧小区，改造旧有居民庭院500个，建设社区休闲健身广场50个，改造土路巷道56万平方米，改善居民社区整体环境。第三，加快改造供水、排水、供气、供热等地下管网设施，强化运营监管，提高基础设施运行的安全性和可靠性。第四，大力实施公交优先战略，推进公交枢纽、场站建设，增加、调整线路10条，新增、更新城市公交车辆300台，加快构建市区公交专用道网络，提高公交覆盖面和运营能力，方便群众出行。第五，严格落实国家和省“稳价保供”政策，强化市场监管，严厉打击囤积居奇、哄抬物价等违法行为。突出抓好“菜篮子”、“米袋子”、“油瓶子”，加大价格调节基金投放力度，建立物价上涨对低收入群体的动态补贴救助机制，努力保障人民群众的基本生活。

　　二是继续扩大就业。大力推进创业型城市建设，落实就业和创业政策，积极发展劳动密集型产业、商贸流通业、家庭服务业和小型微型企业，多渠道开发就业岗位。强化人力资源市场配置，加强高校毕业生、农民工和城镇就业困难人员等特殊群体就业援助，全年开发公益性岗位5000个，培训农民工20万人。

　　三是提高社会保障水平。着重扩大城镇基本医疗保险覆盖面，实现“应保尽保”。把国有困难企业、集体困难企业未参保退休人员纳入城镇职工医疗保险范畴，参保的城镇居民纳入门诊就医统筹金支付范围，增设城镇居民基本医疗保险缴费档次，提高城镇低保和重度残疾人员医疗待遇水平。调整城镇居民失业保险政策，提高失业保险金标准。抓好新农保试点，把主城6区及2县（市）纳入试点范围。完善低保动态调整、核查机制，提高城乡低保标准和低收入家庭救助水平。启动市救灾物资储备库建设，创建国家级标准减灾示范社区30个。

　　四是加快保障性住房建设。围绕构建多层次住房保障体系，推进利用住房公积金贷款支持保障性住房建设试点，全年新增廉租住房受益户2000户，提供经济适用住房4000套，开工建设公共租赁住房1万套，回迁棚改居民2.6万户。落实国家房地产宏观调控政策，加强房地产市场监管，促进房地产业平稳健康发展。

　　五是优先发展教育事业。合理配置教育资源，全面提高教育质量，努力促进教育公平。新建中小学校4所，改造薄弱学校8所，为2.5万名农村寄宿制学校学生、1000名特殊教育学校学生、3万名中等职业学校学生、1.3万名家庭困难农民工子女发放助学金。加快发展学前教育，提高公办幼儿园比例。积极发展职业教育、民族教育、特殊教育、民办教育、高等教育、继续教育，不断完善终身教育体系。

　　六是完善医疗卫生服务体系。深化医药卫生体制改革，完善新农合政策，提高补偿水平，参合率保持98%以上。加快医疗卫生服务体系建设，重点推进7个市属医疗卫生机构项目和10个县级医院项目建设。推进基层医疗卫生体制综合配套改革，抓好公立医院改革试点。加强食品药品监管工作。强化疾病预防控制，提高疾病监测预警和应急处理能力。抓好流动人口计划生育基本公共服务均等化试点。

　　七是大力繁荣文化体育事业。加强乡镇文化基础建设，推进文化惠民工程，依法保护历史文化遗迹、遗址和非物质文化遗产。加快文化市场综合行政执法改革。广泛开展全民健身运动，努力提高市民健康水平。以八区体育场综合改造和乌吉密滑雪场设施完善为突破口，加快体育基础设施建设，打造方便市民的15分钟健身圈，安装健身路径1400件。推进冰雪体育强市建设，提升竞技体育水平。

　　（九）着力加强民主法制建设和社会管理工作，确保社会和谐稳定。稳定是经济发展、社会进步的基本前提和根本保障。我们将认真落实稳定是硬任务、是第一责任的要求，切实加强和改进新形势下群众工作，深入化解社会矛盾，为我市更好更快更大发展创造良好的社会环境。

　　一是加强民主法制建设。认真执行市人大及其常委会的各项决议、决定，依法接受市人大及其常委会的法律监督和工作监督，自觉接受市政协的民主监督。及时办理人大代表建议和政协提案，健全与民主党派、工商联、无党派人士的联系制度。规范行政行为，增强行政工作透明度，畅通公民参政议政渠道，保障公民知情权、参与权、表达权和监督权。加强村（居）委会民主自治和民主管理，依法组织全市第九届村委会换届选举。坚持依法治市、依法行政、公正文明执法，努力创造公平正义的法制环境。

　　二是加强社会稳定工作。深化“平安哈尔滨”创建活动，重点启动实施“3531”工程二期建设，完善社会治安打防管控体系，依法严厉打击各类严重刑事犯罪和涉众型经济犯罪。进一步加强隐蔽战线斗争。切实提高突发事件预防应急处置能力，增强群众安全感。认真落实各级安全生产责任，全面排查安全隐患，加强道路交通、城市消防、危化产品、煤矿和非煤矿山、特种设备、建筑工地监管，有效预防重特大安全事故。加强信访工作，发挥社会组织作用，及时解决、妥善处理人民群众反映的突出问题，最大限度地从源头预防和化解社会矛盾。

　　三是加强精神文明建设。围绕推进新战略和创建“三优”文明城市，实施提升市民文明素质5年培训计划，培育壮大志愿者队伍，扎实推进群众性精神文明创建活动，不断提升城市文明程度和市民文明素质。加强社会公德、职业道德、家庭美德、个人品德建设，营造超越自我、奋发进取、理性平和、友善诚信、邻里和睦、开放包容的社会氛围。加强未成年人思想道德建设，完善学校、家庭、社会“三结合”教育网络。制定并实施《哈尔滨市妇女发展规划（2011-2015年）》和《哈尔滨市儿童发展规划（2011-2015年）》，推进妇女儿童事业健康发展。深入开展拥军优属、拥政爱民活动，巩固扩大“全国双拥模范城”成果。

　　四是加强城乡社区建设。开展“星级和谐社区”创建活动，促进家庭和谐、邻里和谐，带动社会和谐。推广“中心社区”建设模式，建成使用面积超千平方米中心社区5个。继续推进社区公益用房达标建设，确保主城区社区公益用房平均使用面积300平方米以上。统筹推进农村社区建设，力争启动率达到70%。

　　（十）着力加强政府自身建设，切实提高行政效能和服务水平。转变政府职能，增强行政效能，提高服务水平，是实施新战略、实现新目标的重要保证，也是新形势、新任务对政府工作提出的新要求。我们将认真履行人民赋予的权力，切实增强责任感和使命感，努力营造风清气正的行政环境、宽松和谐的发展环境，明显提升政府的公信力和社会满意度。

　　一是进一步超越自我。把超越自我作为加强公务员思想作风建设的重要内容，深入开展“做主人、敢担当、谋发展、惠民生”学习讨论活动。要求各级领导干部把事业当家业、把工作当学问，率先垂范、自加压力，用宽视野、高站位谋划发展，用实举措、强手段推动发展，把惠及民生的各项任务落到实处。教育广大公务员深化市情认识，看到差距、正视不足，切实增强迎难而上、敢于担当的勇气和信心，努力营造竞相超越自我、创先争优的良好氛围。

　　二是扎实开展“政风建设年”活动。针对部分公务员存在的服务意识差、办事效率低、业务能力弱以及纪律松懈、推诿扯皮、吃拿卡要等突出问题，重点对91个具有审批、执法和服务职能的部门、窗口和关键部位进行监测，对50个涉及全市发展战略、投资额在亿元以上大项目的落实情况进行全程跟踪。通过自查自纠、设立专门网站、开设投诉电话等多种渠道，深入查找和剖析问题，从严进行集中整治，切实解决一些政府部门“门难进、脸难看、事难办”等问题。开展“百家企业、百名外商、百位代表委员评政风”活动，加大明察暗访力度，强化对政府部门的全方位监督。有针对性地建立健全长效管理机制，努力实现政风建设的常态化、制度化，着力打造素质过硬、作风优良的公务员队伍，树立服务型政府的良好形象。

　　三是不断提高执行力和推动力。坚持把创新作为提高执行力的“倍增器”，积极探索改进工作、解决问题、提高效率的新举措，努力实现行政效能最大化。坚持把调查研究作为谋事之基、成事之道，深入实际，贴近群众，及时掌握社情民意，完善决策风险评估机制，提高决策的科学化、民主化水平。大力倡导雷厉风行、求真务实的工作作风，进一步精文减会，实行重点工作包保责任制，督促各级领导干部靠前指挥、靠前服务，把更多的时间和精力用在抓发展、促落实上。建立健全有效的横向协调机制，完善市直部门与区县（市）工作联动机制，提高政府综合调控能力，着力解决条块分割、相互掣肘、互为障碍的问题，确保政令畅通。按照“权随事走”的原则，加快政府职能转变，理顺政府机构设置和职责分工，进一步下放事权，为人民群众提供便捷、高效、满意的服务。

　　四是努力改善经济发展环境。牢固树立“企业创造财富、政府创造环境”的理念，进一步深化行政审批制度改革，建立标准一体化、环节整体化、进度同步化的审批新机制。对重大项目实行代办制，为投资者提供周到、快捷的无障碍服务。全面推行政府绩效管理，深入推进政务公开，严格行政问责，凡是把项目推走、挤跑、拖垮，甚至损害人民群众切身利益的公务员，都要坚决问责，严肃处理。

　　五是切实加强廉政建设。全面贯彻落实中纪委十七届六次全会精神，以完善惩治和预防腐败体系为重点，整体推进反腐倡廉各项工作。大力弘扬勤俭节约、艰苦奋斗的优良作风，做到公务用车、会议、公务接待、党政机关人员出国四项经费支出“零增长”。着力解决人民群众反映强烈的突出问题，坚决纠正对群众疾苦漠不关心、对群众呼声置若罔闻、对群众利益麻木不仁的官僚行为。突出抓好对工程建设、征地拆迁、产权交易、政府采购等重要领域和关键环节的监督，坚决查处与民争利、以权谋私的腐败行为，始终保持惩治腐败的高压态势，把实施新战略的各项工程建设成为经得起检验的廉政工程、德政工程、民心工程。

　　各位代表，哈尔滨又一次站在新的历史起点，前景光明，任重道远。让我们更加紧密地团结在以胡锦涛同志为总书记的党中央周围，在省委、省政府和市委的坚强领导下，同心同德、凝神聚力，奋发有为、开拓进取，以经济社会更好更快更大发展的优异成绩，迎接中国共产党成立90周年！
